# Contact Lens Safety for the Correction of Refractive Error in Healthy Eyes

**DOI:** 10.1097/ICL.0000000000000938

**Published:** 2022-06-10

**Authors:** Michelle K. Rhee, Deborah S. Jacobs, Deepinder K. Dhaliwal, Loretta Szczotka-Flynn, Christina R. Prescott, Vishal Jhanji, Thomas L. Steinemann, Bruce H. Koffler, Bennie H. Jeng

**Affiliations:** Department of Ophthalmology (M.K.R.), New York, NY, Icahn School of Medicine at Mount Sinai, NY, NY; Department of Ophthalmology (D.S.J.), Boston, MA, Massachusetts Eye and Ear, Boston, MA; Department of Ophthalmology (D.K.D.), Pittsburgh, PA, University of Pittsburgh School of Medicine, Pittsburgh, PA; Department of Ophthalmology (L.S.‐F.), Cleveland, OH, Case Western Reserve University, Cleveland, OH; Department of Ophthalmology (C.R.P.), New York, NY, NYU Grossman School of Medicine, NY, NY; Department of Ophthalmology (V.J.), Pittsburgh, PA, University of Pittsburgh School of Medicine, Pittsburgh, PA; Department of Ophthalmology (T.L.S.), Cleveland, OH, Case Western Reserve University, MetroHealth Division of Ophthalmology, Cleveland, OH; Department of Ophthalmology (B.H.K.), Huffman and Huffman, Lexington, KY, Lexington, KY; and Department of Ophthalmology (B.H.J.), Baltimore, MD, University of Maryland School of Medicine, Baltimore, MD.

**Keywords:** Contact lens, Medical device, Keratitis

## Abstract

Contact lenses are a safe and effective method for correction of refractive error and worn by an estimated 45 million Americans. Because of the widespread availability and commercial popularity of contact lenses, it is not well appreciated by the public that contact lenses are U.S. Food and Drug Administration (FDA)–regulated medical devices. Contact lenses are marketed in numerous hard and soft materials that have been improved over decades, worn in daily or extended wear, and replaced in range of schedules from daily to yearly or longer. Lens materials and wear and care regimens have impact on the risks of contact lens–related corneal inflammatory events and microbial keratitis. This article reviews contact lens safety, with specific focus on the correction of refractive error in healthy eyes.

Eye care providers know that it is critical to thoroughly counsel patients regarding risks of surgery, including refractive surgery, before any planned intervention. However, even experienced eye care providers may not adequately discuss the risks of contact lens use, including inflammatory events and infection. Although one might presume that contact lens wear is safer than refractive surgery, it has been argued that infection may be eight times higher with lifetime daily contact lens use compared with laser-assisted in situ keratomileusis (LASIK).^1^ However, more recently, Wu et al^2^ reported that daily wear contact lenses and extended wear silicone hydrogel contact lenses need to be worn for 103 (95% CI 103–391) and 25 (95% CI 25–79) years, respectively, to equal the rate of vision loss equivalent to a one-off LASIK procedure. Despite the generally low risk of vision loss with contact lens wear or refractive surgery, vision-threatening complications do occur. Approximately 45 million (or nearly 14% of the population) Americans wear contact lenses^3^ that comprises the number one risk factor for microbial keratitis.^4^

As contact lens technology evolves, more people are using contact lenses for indications beyond the correction of refractive error. Decorative contact lenses are available both with and without optical correction, and decorative contact lenses may be used routinely or for specific occasions such as costume parties or Halloween. Soft toric contact lenses are available to individuals with astigmatism who previously wore rigid gas permeable (RGP) lenses. Multifocal lenses are an option for presbyopic individuals who might otherwise have stopped wearing contact lenses once reading glasses were needed. A soft lens (MiSight 1 day; CooperVision, Inc., Victor, NY) has been U.S. Food and Drug Administration (FDA) approved for slowing the progression of myopia, potentially increasing the use of contact lenses in pediatric patients. Orthokeratology (OK) is also being used in an off-label fashion for myopia control, resulting in more overnight contact lens use in younger patients.^5^ New contact lens technology allows continuous drug delivery to the ocular surface.^6^ In the near future, contact lenses may be used to monitor eye pressure for patients with glaucoma^7^ and track glucose control for diabetic patients.^8^

Ophthalmologists and optometrists must stay up to date on the advances in contact lens technology to best inform, advise, and educate our patients. Ideally, all contact lenses should be fitted and prescribed by an eye care professional, after a comprehensive eye examination to exclude underlying disease, and after discussion about the risks and benefits of the various lens options. The prescribing clinician should also attend to education and training as to proper wear and care and supervise wear with appropriate follow-up. Unfortunately, patients may choose to obtain contact lenses online (such as www.pinkyparadise.com) or through a nonophthalmic retail provider, without an examination or adequate education. This is especially a problem with decorative contact lenses, which may be sold over the counter, and are not well-regulated.^9^ Public education regarding contact lens safety is critical because some patients may unwittingly put themselves at risk by wearing contact lenses without the guidance of an eye care professional. This article reviews contact lens safety, with specific focus on the correction of refractive error in healthy eyes.

## CONTACT LENSES ARE MEDICAL DEVICES

All contact lenses, regardless of intended use, are U.S. FDA–regulated medical devices. Before a 2005 Senate bill that upheld this statement, there was a legal loophole that allowed for decorative plano contact lenses to be free from the medical device categorization on the basis that their use was intended solely to change eye appearance.^10^ Because of the infections that resulted from the use of unregulated and unsupervised decorative lenses, the American Academy of Ophthalmology, American Academy of Optometry, and Contact Lens Association of Ophthalmologists (now known as Eye and Contact Lens Association) convened with the FDA in 2002, and in conjunction with advocacy led by Thomas L. (Tim) Steinemann MD, an amendment to the Federal Food, Drug, and Cosmetic Act (public law 109–96) was signed into law on November 9, 2005.

Although contact lenses are a safe and effective option for the correction of refractive error, the contact lenses are medical devices that come with risk. Contact lens–related disorders carry a significant burden in the United States. Collier et al^4^ reported 2010 data from the United States national outpatient and emergency department database that revealed nearly one million clinical visits for all types of contact lens–related keratitis with a cost of $175 million. The number one cause of medical device–related pediatric emergency department visits was related to contact lens.^11^

There are three FDA medical device classes based on the risks associated with the device, with class I being low-risk devices such as adhesive bandages. Daily wear soft contact lenses are class II, whereas extended wear soft contact lenses and overnight OK lenses are class III that acknowledges their high risk of microbial keratitis. Examples of other class III devices include intraocular lenses, breast implants, and pacemakers. The FDA has an online site called MedWatch, where physicians and patients can voluntarily report problems with medical devices. Medical device manufacturers are required to report adverse events to MedWatch, such as those involving contact lenses, intraocular lenses, or donor cornea tissue. The Centers for Disease Control and Prevention (CDC) uses data from MedWatch to review contact lens–related infections.^12^ Contact lens–related adverse events have been underreported because of the voluntary nature of MedWatch for clinicians and patients. The CDC urges prompt reporting of serious adverse events to MedWatch to help the FDA identify and understand the risks of contact lenses especially because there is no other registry or formal surveillance of contact lens–related morbidity in the United States.^3^

## CONTACT LENS WEAR SCHEDULE, REPLACEMENT SCHEDULE, AND MATERIALS

Wear schedule (daily wear vs extended wear), replacement schedule (daily disposable vs weekly, two weekly, monthly, quarterly, or yearly replacement), and materials (silicone hydrogel vs hydrogel) are the major considerations in soft contact lens wear.

Overnight extended wear of contact lenses has been associated with manyfold increased risk of contact lens–related keratitis when compared with daily wear.^13,14^ Separate from wear schedule (extended vs daily wear), there is evidence that a more frequent-replacement schedule is advantageous. Solomon et al^15^ found in their cohort study that daily disposable contact lens wearers were more likely to be asymptomatic; have better vision and overall satisfaction; and experience fewer complications, lens surface deposits, and less redness and cloudy vision when compared with reported outcomes for conventional daily wear groups. Suchecki and associates^16^ also found that daily disposable contact lens wearers experienced less complications, as defined by “events per person year.” Muhafiz and team^17^ showed that when compared with reusable hydrogel contact lenses, daily disposable hydrogel contact lenses caused less inflammation, as measured by IL-6, IL-8, and IL-17A in the tear film.

When comparing complications related to contact lens replacement schedule, one confounding factor that must be considered is that the patient replacement schedule is not always the same as the manufacturer recommended schedule. Noncompliance with recommended wear and replacement schedules is a common problem; a 2016 CDC study reported that 24% to 52% of contact lens wearers replace their lenses at intervals longer than prescribed.^18^ In a study by Rueff et al,^19^ patients who used daily disposable contact lenses showed a noncompliance rate of 6.1%, whereas patients who used monthly disposable contact lenses showed a noncompliance rate of 33.9%, and patients who used 2-week disposable contact lenses showed a noncompliance rate of 60%. Unfortunately, in this same study, the most commonly prescribed replacement schedule was biweekly (45.5%), followed by monthly (34.3%), with daily replacement schedule being the least common (20.2%). Patients most commonly report “forgetting which day to replace lenses” as the reason for noncompliance with 2-week and monthly replacement schedules and “to save money” as the reason for noncompliance with daily disposable schedules.^20^

It is unclear why patients who follow a biweekly replacement schedule have a higher rate of noncompliance than those who follow other replacement schedules, but eye care providers may be partly to blame. Dumbleton et al.^21^ reported 18% (United States) to 34% (Canada) of eye care providers recommended replacement intervals longer than those recommended by the manufacturer for 2-week replacement lenses. This is in comparison with 4% (United States) to 6% (Canada) of eye care providers who recommended replacing daily disposable lenses less frequently than recommended, and 1% (United States) to 2% (Canada) who recommended extending the monthly wear schedule.^21^ In a follow-up study, Dumbleton et al.^22^ surveyed patients from Australia, the United Kingdom, the United States, and Norway; the issue of noncompliance with replacement schedule was discovered to be present in all countries but was highest in Australia and lowest in Norway.

## LENS CARE AND LENS STORAGE CASES

It is well-established that lens storage case contamination is a risk factor for microbial keratitis (see below) and that daily disposable lens wear has lower risk of severe or sight threatening disease compared with frequent-replacement hydrogel lenses.^23,24^ For these reasons, daily disposable soft lens wear and daily wear of rigid corneal lenses can be considered the safest modes of contact lens wear.^25^

A patient may require a reusable lens if the lens parameters needed for good fit and refractive correction are not available in a daily disposable design. Reduced cost may also drive some patients toward planned replacement over daily disposable lenses, although the gap in cost is narrowing and one must factor in the cost of solutions and cases in the calculation of annual cost. Options for overnight disinfection include the use of multipurpose solution (MPS) or a peroxide system. Peroxide systems are not as convenient as MPS but are associated with lower risk of both infectious^26^ and inflammatory complications^27^ and have the highest microbicidal activity.^28–30^

If a patient is using reusable lenses, appropriate choice and use of solutions, case hygiene, and frequent case replacement and must be emphasized to reduce the likelihood of infections.^24,31^

## CORNEAL INFILTRATIVE EVENTS

**FIG. 1.**
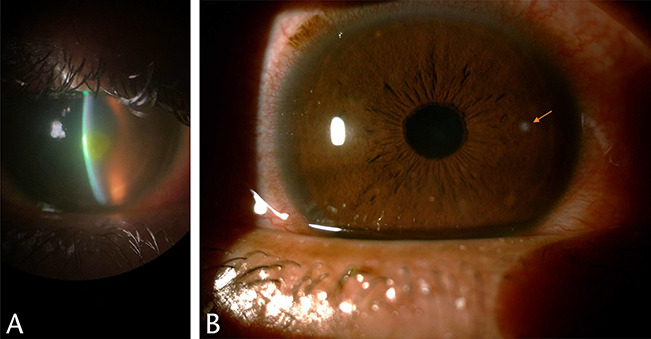
(A) (Michelle Rhee, MD). Paracentral corneal infiltrate caused by *Pseudomonas aeruginosa* secondary to contact lens wear. (B) (Thomas Steinemann, MD). Daily disposable contact lens use–related small corneal infiltrate.

A well-established risk of soft contact lens wear includes corneal infiltrative events (CIEs). In daily, reusable, soft lens wear, the annual incidence of symptomatic CIEs is approximately 3%. The incidence of symptomatic CIEs during extended soft lens wear ranges from 2.5% to 6%; when asymptomatic CIEs are included, the incidence can be as high^32^ as 20% to 25% (Fig. 1A,B). The epidemiology of CIEs shows the incidence rates by wear schedule (daily wear vs overnight use), material (silicone hydrogel vs hydrogel), or disposal frequency (daily disposable vs reusable).^33^ In addition to overnight (extended) wear increasing the incidence of microbial keratitis,^24,34^ extended wear increases the risk of CIEs as well.^35–39^ The literature documents a 2× to 7× high risk for CIEs with extended versus daily wear.^35,37,38^ Although not clear why, patients aged 15 to 25 years have the greatest risk of CIEs, with children having a relatively lower rate of CIEs.^35^ Regarding lens material, despite the known physiological advantages of silicone hydrogel lenses compared with traditional hydrogel lenses with respect to high oxygen transmissibility, the risk of CIEs is high with reusable lenses silicone hydrogel lenses. The relative risk of a CIE is approximately 2-fold for reusable silicone hydrogel lenses versus hydrogel materials, which persists regardless of mode of wear (daily or extended wear).^35,38,40–42^

In disposal frequency, daily disposable lenses have been found to dramatically reduce the incidence of CIEs compared with reusable lenses.^13,39^ One study prospectively assessed the incidence of CIEs with silicone hydrogel and nonsilicone hydrogel daily disposable lenses and found a near-zero incidence of CIEs, with a rate of 0.4% (95% CI 0.1%–1.5%) per year in silicone hydrogel daily disposables and 0% in hydrogel daily disposables.^33^ The large reduction of CIEs in daily disposable lens wear is considered to be driven by a reduction of lens-associated bioburden—including lack of bioburden from not requiring the use of a lens storage case in daily disposable lens wear.^43^ In fact, continual reuse of lens storage cases, presumably resulting in greater storage case contamination, results in approximately an 8-fold greater risk of CIEs.^39^

## CONTACT LENSES AND TAP WATER SHOULD NOT MIX

**FIG. 2.**
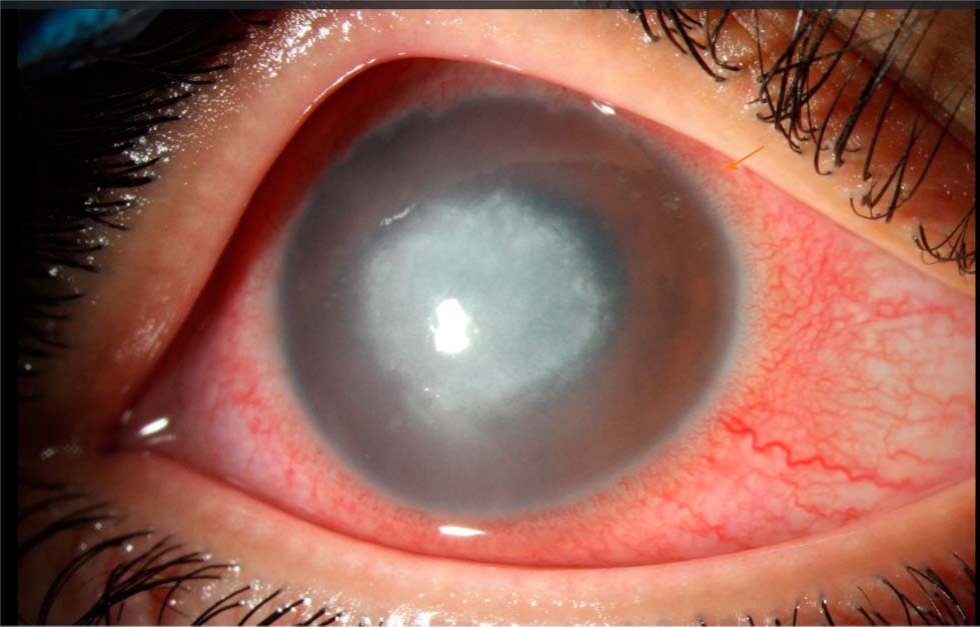
(Rebecca Rojas, OD). Slitlamp photograph showing *Acanthamoeba* keratitis related to decorative contact lens use.

Tap water exposure during contact lens wear or cleaning can result in inflammatory^44^ or microbial^45^ keratitis. One particularly devasting microbial corneal infection is caused by *Acanthamoeba* spp (Fig. 2). Contact lens wear may predispose the cornea to a high risk of microbial invasion,^46^ and amoebae present in water can gain access to the cornea where amoebae can multiply and cause an infection that is difficult to eradicate. There is often a delay in *Acanthamoeba* diagnosis because the early infection can be mistaken for herpes simplex keratitis: the average time to diagnosis is 27 days.^47^ *Acanthamoeba* exist in an active trophozoite form, but then encyst when exposed to harsh environments. The cyst form is more resistant to anti-amoebal therapy. Multipurpose contact solutions have been mainly ineffective against *Acanthamoeba*,^48^ although heat disinfection or two-step hydrogen peroxide can be cysticidal.

“Water exposure” includes not only storing and rinsing contact lenses in tap water but also includes showering or swimming with contact lenses. Although the risks of water exposure and contact lens use have been known for decades, this behavior prevails.^49^ A recent study shows most soft contact lens wearers, and nearly all RGP contact lens wearers, regularly expose their lenses to water, many unaware of the risk. Specifically, 86% of soft and 67% of RGP lens users wear lenses when showering, and 62% of soft and 51% of RGP lens users wear lenses when swimming.^49^

Patients must also take care to disinfect their reusable lenses properly by “rub-and-rinse” with care product (not tap water) to reduce microbes, avoiding “topping off” of care product solutions, never reusing old solution or using tap water as the storage media in cases, replacing storage cases every 3 months, drying with a clean tissue, and storing upside down with the caps off after each use.^4^

Contact lens wearers are not entirely at fault. Manufacturers play a part in the confusion as to the ideal way to care for contact lenses. In a survey of contact lens solutions available in the United States, none of the products for soft contact lenses recommended use of tap water, but alarmingly, 83% of products related to RGP contact lenses recommended the use of tap water to rinse surfactant off of contact lenses and/or the storage case.^50^ The Eye and Contact Lens Association advocated for change regarding these instructions; this resulted in a successful change in the labelling of contact lens solution to eliminate use of tap water for a manufacturer of contact lens solution.

## EXTENDED WEAR CONTACT LENSES

**FIG. 3.**
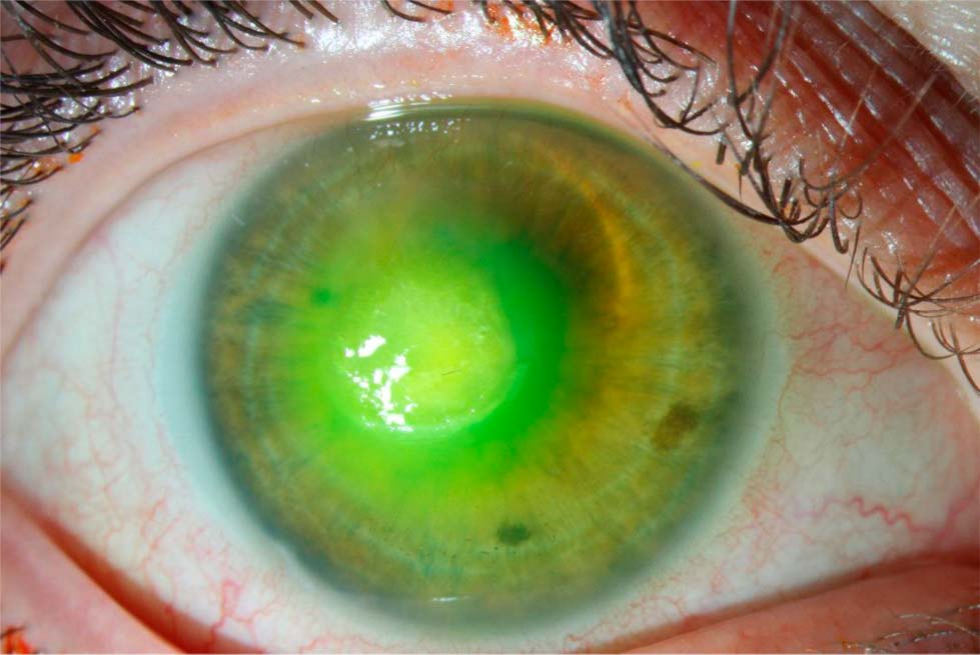
(Rebecca Rojas, OD). *Pseudomonas aeruginosa* contact lens–related keratitis in a patient who slept in extended wear contact lenses.

Extended wear contact lenses can be used continuously for a period of one to four weeks. Clearly, this is a convenient and hence a popular method of contact lens use. However, there are concerns regarding the safety of extended wear contact lenses. Lim et al.^51^ noted that the incidence of microbial keratitis in daily wear has been estimated at two to five per 10,000 lens wearers per year, whereas extended wear was found to have a 10- to 15-fold increased risk, and any overnight wear led to an 8-fold excess risk. Figure 3 shows a large, central corneal infiltrate secondary to *Pseudomonas aeruginosa* infection in a patient with extended wear contact lens–related keratitis.

The perils of overnight wear were demonstrated when hydrogel lenses were worn for extended wear in the early 1990s. The incidence of microbial keratitis was as high as 13.3 to 20.9 per 10,000 wearers per year for extended wear compared with 2.2 to 4.1 per 10,000 for daily wear and two per 10,000 for rigid lenses.^52^ Silicone hydrogel lenses were developed to increase the oxygen transmissibility (Dk/t) with the hypothesis that better maintenance of the integrity of the corneal epithelium would reduce the incidence of microbial keratitis. However, the first cases^53^ of microbial keratitis in silicone hydrogel extended wear contact lenses were reported in 2002, with further analysis making clear that lens material itself is not protective against microbial infection.^54^ Schein et al.^42^ reported that the incidence of loss of visual acuity because of microbial keratitis among users of the silicone hydrogel contact lens was low.

## DECORATIVE CONTACT LENSES

Decorative (colored, cosmetic) lenses pose significant (up to 16.5×) microbial keratitis risk compared with corrective contact lenses.^55^ Major factors that contribute risk include: unregistered/unregulated manufacture, distribution, and sale, leading to wearers obtaining lenses without professional guidance. Often there is no assessment, fitting, prescription, or counseling/aftercare because the sale is often “over-the-counter” from unlicensed vendors.^56,57^ Selling contact lenses without a doctor's prescription is illegal in many countries, including the United States. In bypassing legal regulatory safeguards, dangerous unapproved products may be sold to unsuspecting, contact lens-naive wearers; inappropriate lens wear and care often result.^56^ Likewise, it is difficult to assess and appropriately monitor these younger, inexperienced users, who frequently have not been instructed on proper storage and safe handling. The incidence of use and disease in these populations is unknown. Sauer et al.^30^ cited cosmetic contact lens wear as an independent risk factor (odds ratio 1.37; 95% CI 1.14–1.65) for microbial keratitis (compared with corrective lenses) in a multicenter case–control study.

Surveys indicate frequent online (24%) purchase or borrowing/obtaining (18%) from friends and family.^58^ In this survey, most unauthorized sellers reviewed did not adhere to the proper protocol for selling contact lenses or instruct their customers on proper lens wear and care.^58^ This might predispose the users to contact lens–related complications.

Some risks may be related to the lenses and their properties: roughened surfaces (paint, dyes, and pigment) can irritate the ocular surface and act as a binding reservoir for microbial organisms.^59^ Some lenses may be illegally seized and repackaged, even sold as counterfeit: these lenses are associated with high (60%) rates of contamination.^60^ Other risky lens properties include more lens thickness, poor oxygen transmissibility, and low water content, all of which can contribute to ocular surface instability and disease.^8^

Decorative contact lens users are more likely to be young and contact lens-naïve first-time wearers. Recent surveys on decorative lens wearers indicate frequent use among teens and young women (14%–24% of respondents).^61,62^ Over 70% of respondents reported first use at less than 15 years of age.^61^ Despite young wearers' frequent usage, related knowledge about decorative contact lenses is low. Half of users obtained lenses without prescription from unlicensed vendors.^61,62^ Because single-use lenses sometimes cost more, the likelihood is high that wearers will choose cheaper (reusable) lenses, even though colored lenses are available for daily disposable use. Stapleton et al^63^ performed a large, prospective, multicenter study exploring cosmetic contact lens–related corneal infections in Asia. Cosmetic contact lens infections comprised 7% to 54% of cases across the region. Cosmetic contact lens wearers were more likely to be female and younger than 25 years, have a shorter period of wear experience, and wear lenses of hydrogel materials with added pigment on the back surface. There was a higher rate of *Acanthamoeba* infections (9%) in cosmetic wearers compared with refractive wearers (1%).

## ORTHOKERATOLOGY

We include here a section on OK because of its increasing relevance and popularity as a treatment option for myopia control in the emerging myopia epidemic. In carefully selected patients with close supervision and follow-up, overnight wear used for the slowing of myopia progression may be considered, with the clinician and patient and family understanding that this is off-label use of a medical device. As an alternative, use of the daily disposable, MiSight 1 day soft lens (CooperVision, Inc., Victor, NY), the only FDA-approved contact lens for myopia control, might be considered.

Acceptance of the efficacy of OK in slowing myopic progression is complicated by its overnight mode of wear. OK uses specially designed, reverse geometry, RGP contact lenses to temporarily reshape the corneal surface.^64^ These lenses flatten the cornea and are worn overnight to treat myopia. Most OK wearers are young children being fit for myopia control.

Between 2001 and 2006, there were reports from southeast Asia of OK-related microbial keratitis, several of which were caused by *Pseudomonas* and *Acanthamoeba*.^65^ Investigations into these reports showed a lack of patient compliance with disinfection and the use of tap water, along with the absence of regulation in fitting practices and inappropriate lens materials or lens designs. More recent worldwide studies have shown OK to be safe with rare complications being reported when performed by well-trained practitioners.^66^ Bullimore et al. ^67^ did a large retrospective study in the United States involving 1,317 patients, representing 2,600 patient-years. There were only two cases of microbial keratitis noted, both of which occurred in children, but neither resulted in a loss of visual acuity. The overall incidence of microbial keratitis was 7.7 per 10,000 patient-years (95% CI 0.9–27.8), and specifically for children, overall incidence was 13.9 per 10,000 patient-years (95% CI 1.7–50.4) of wear.^67^

Overnight OK lenses were FDA approved in 2002 to treat myopic refractive error, but not for myopia control, and therefore are used off-label for the latter purpose. Although OK seems to be effective in myopia control at a similar level as atropine,^68^ the safety concerns with overnight wear must be taken into consideration.

## DISCUSSION

Contact lenses, when worn and cared for properly, are a safe and effective method for correcting refractive error. Among the variety of lenses, daily disposable contact lenses are more likely to be prescribed and worn as recommended by the manufacturer and have significantly lower risks of complications, including inflammation and infection. However, despite these advantages, it remains the case that in the United States, the daily disposable contact lenses represent only approximately 50% of lenses prescribed, with the other half composed of biweekly and monthly contact lenses.^69^ This is probably because of the perception that daily disposable contact lenses are more expensive. However, sight threatening ocular infections carry a high cost to the patient and society^4,70^ and should be considered by the provider and an informed patient before making decisions based on lens cost.

There is substantial data cited above to support the recommendation that daily disposable mode of wear is the safest of all modes of soft contact lens wear. The advantages of daily disposable mode of wear outweigh any advantage conferred by specific type of soft lens material. It is our position that if soft lens wear is being considered, daily disposable lenses should be prescribed whenever possible.

In some cases, the visual needs of the patient cannot be met by lenses marketed for daily disposable wear. In such cases, yearly replacement, programmed replacement, or frequent-replacement lenses may be necessary. Such lenses may carry regulatory approval for overnight or extended wear, but overnight or extended wear is associated with high risk of both infectious and inflammatory complications. For this reason, unless the specific benefit of overnight wear exceeds the risks, we recommend against overnight or extended wear. For patients for whom a scheduled replacement soft lens is the only option for satisfactory fit and correction of refractive error, peroxide disinfection is the preferred disinfection mode rather than the use of MPS. This is particularly so if a patient has a tendency to hypersensitivity or allergy or if there is history of infiltrative or microbial keratitis. Furthermore, patients with tendency toward inflammation or CIEs may do better in hydrogel rather than silicone hydrogel lenses. Any patient-prescribed reusable lenses should receive instruction in best practices as far as solutions, lens care, and case hygiene.

Although the FDA acknowledges the higher risk of extended wear contact lenses by its class III medical device designation, it is not clear to patients that this distinction exists. One way to influence additional labelling of risk is for doctors and patients to report all CIEs and contact lens–related infections to MedWatch. Patients probably perceive contact lenses as a benign commodity or beauty product available through online shopping rather than as a medical device. Patients are often surprised to hear that extended wear is risky. There is a clear need for consistency and clarity of messaging. Given the limited safeguards and ease of online purchase domestically and globally, education and supervision of our patients is more critical than ever. The harmonization of counseling, labelling, and messaging from physicians, industry, and governmental organizations is of paramount importance for contact lens safety.
